# Genetic diversity and population structure of indigenous chicken in Rwanda using microsatellite markers

**DOI:** 10.1371/journal.pone.0225084

**Published:** 2020-04-02

**Authors:** Richard Habimana, Tobias Otieno Okeno, Kiplangat Ngeno, Sylvere Mboumba, Pauline Assami, Anique Ahou Gbotto, Christian Tiambo Keambou, Kizito Nishimwe, Janvier Mahoro, Nasser Yao

**Affiliations:** 1 Animal Science and Veterinary Medicine, University of Rwanda, College of Agriculture, Nyagatare, Rwanda; 2 Department of Animal Science, Animal Breeding and Genomics Group, Egerton University, Nakuru, Kenya; 3 Faculty of Agronomy and Biotechnologies, Masuku University of Science and Techniques, Franceville, Gabon; 4 Biosciences eastern and central Africa, International Livestock Research Institute Hub, Nairobi, Kenya; 5 Laboratoire de genomique Fonctionnelle et Amélioration Génétique Université Nangui Abrogoua, Abidjan, Cote d'Ivoire; 6 Faculty of Agriculture and Veterinary medicine, University of Buea, Buea, Cameroon; National Cheng Kung University, TAIWAN

## Abstract

Rwanda has about 4.5 million of indigenous chicken (IC) that are very low in productivity. To initiate any genetic improvement programme, IC needs to be accurately characterized. The key purpose of this study was to ascertain the genetic diversity of IC in Rwanda using microsatellite markers. Blood samples of IC sampled from 5 agro-ecological zones were collected from which DNA was extracted, amplified by PCR and genotyped using 28 microsatellite markers. A total of 325 (313 indigenous and 12 exotic) chickens were genotyped and revealed a total number of 305 alleles varying between 2 and 22 with a mean of 10.89 per locus. One hundred eighty-six (186) distinct alleles and 60 private alleles were also observed. The frequency of private alleles was highest in samples from the Eastern region, whereas those from the North West had the lowest. The influx of genes was lower in the Eastern agro-ecological zone than the North West. The mean observed heterozygosity was 0.6155, whereas the average expected heterozygosity was 0.688. The overall inbreeding coefficient among the population was 0.040. Divergence from the Hardy-Weinberg equilibrium was significant (p<0.05) in 90% of loci in all the populations. The analysis of molecular variance revealed that about 92% of the total variation originated from variation within populations. Additionally, the study demonstrated that IC in Rwanda could be clustered into four gene groups. In conclusion, there was considerable genetic diversity in IC in Rwanda, which represents a crucial genetic resource that can be conserved or optimized through genetic improvement.

## Introduction

Poultry keeping is an agricultural enterprise with a high potential in Rwanda. More than 40% of households keep poultry with indigenous chickens (IC) being the most preferred, accounting for approximately 80% of the reared chicken species [1]. Raising IC is preferred to exotic breeds because of their small cost of production, scavenging capacity and adaptability to harsh environmental conditions. IC production serves a critical role as a source of revenue for resource-limited countryside families [2]. However, the productivity of IC in Rwanda is low. Each mature hen weighs between 0.8 to 1.8 kg and produces an average of 40 to 100 eggs per year. This output is insufficient to meet the needs of the population [3] and mitigate poverty among the smallholder farmers in rural areas. To improve the genetic potential of IC in Rwanda, different crossbreeding programmes between IC and exotic chicken have been initiated. However, these programmes have not been sustainable due to decreased broodiness in the hybridized birds, unpredictable stock, and the high cost of buying and sustaining exotic cocks for breeding purposes. Additionally, recent global efforts to preserve native genetic resources pose a threat to such programmes [4]. There is, therefore, a need for the development of an alternative strategy to genetic improvement and conservation of IC.

Genetic improvement through within-breed selection of IC in Rwanda could be a promising alternative strategy. Nonetheless, genetic enhancements need a resolute breeding objective, sustainable breeding plans, and an in-depth comprehension of the genetic diversity of prevailing genotypes and ecotypes [5]. Therefore, elucidating the genetic characteristics of the prevailing IC stock will not only favor genetic enhancement but will also expedite their preservation [4].

In various parts of the world, the genetic diversity of IC has been assessed using molecular markers including microsatellites [6–19]. Microsatellites are short, tandemly repeated simple sequences with one to six base pairs in length [20]. Thirty (30) microsatellite markers have been suggested by the Food and Agriculture Organization to be used in the evaluation of genetic diversity in chicken [20–21]. These microsatellite markers are appropriate for a wide range of applications and have remained the most commonly used markers in studies of genetic diversity and population structure since the early 1990s [20,22,23] due to their high degree of polymorphism, random distribution across the genome, codominance, and neutrality with respect to selection [24]. Additionally, they are relatively cheaper to genotype and offer more population genetic information per marker than single nucleotide polymorphisms (SNPs) known as biallelic markers [25]. Finally, microsatellites can successfully amplify low DNA concentration or low-quality DNA samples [26].

There is, however, a scarcity of data on the genetic diversity and population structure of IC in Rwanda. The availability of such knowledge could drive the understanding of the origin and genetic variability in the population to guide selection decisions. As a result, it would be possible to develop apposite mating plans to uphold genetic variation and minimize inbreeding in the population, which would promote response to selection. This study evaluated the degree of genetic diversity and phylogenetic relationships between populations of IC in Rwanda using simple sequence repeats (SSR) markers.

## Materials and methods

### Ethical statement

After a thorough review and approval of sampling procedures and experimental manipulations, ethical permission (Ref: 031/19/DRI September 2, 2019) for the collection of chicken blood samples was obtained from the Research Screening and Ethical Clearance Committee of the College of Agriculture, Animal Sciences and Veterinary Medicine, University of Rwanda. Private grounds were never entered without the consent of chicken owners. The owners of the chicken signed an informed consent form to allow collection of blood sample from their chicken to be used for the experiment. A memorandum of understanding between University of Rwanda, Rwanda Agriculture and Animal Resources Development Board and Ministry of Agriculture had been made to oversee research and consent research activities including procedures to be undertaken in the whole country. Therefore, no specific permissions were needed for each location visited. Every zone was visited in a company of Rwanda Agriculture and Animal Resources Development Board employee who ensured that national and international guidelines were followed. In addition, the chickens were treated humanely, and none of them was sacrificed for this study.

### Collection of samples and DNA extraction

**Fig 1:**
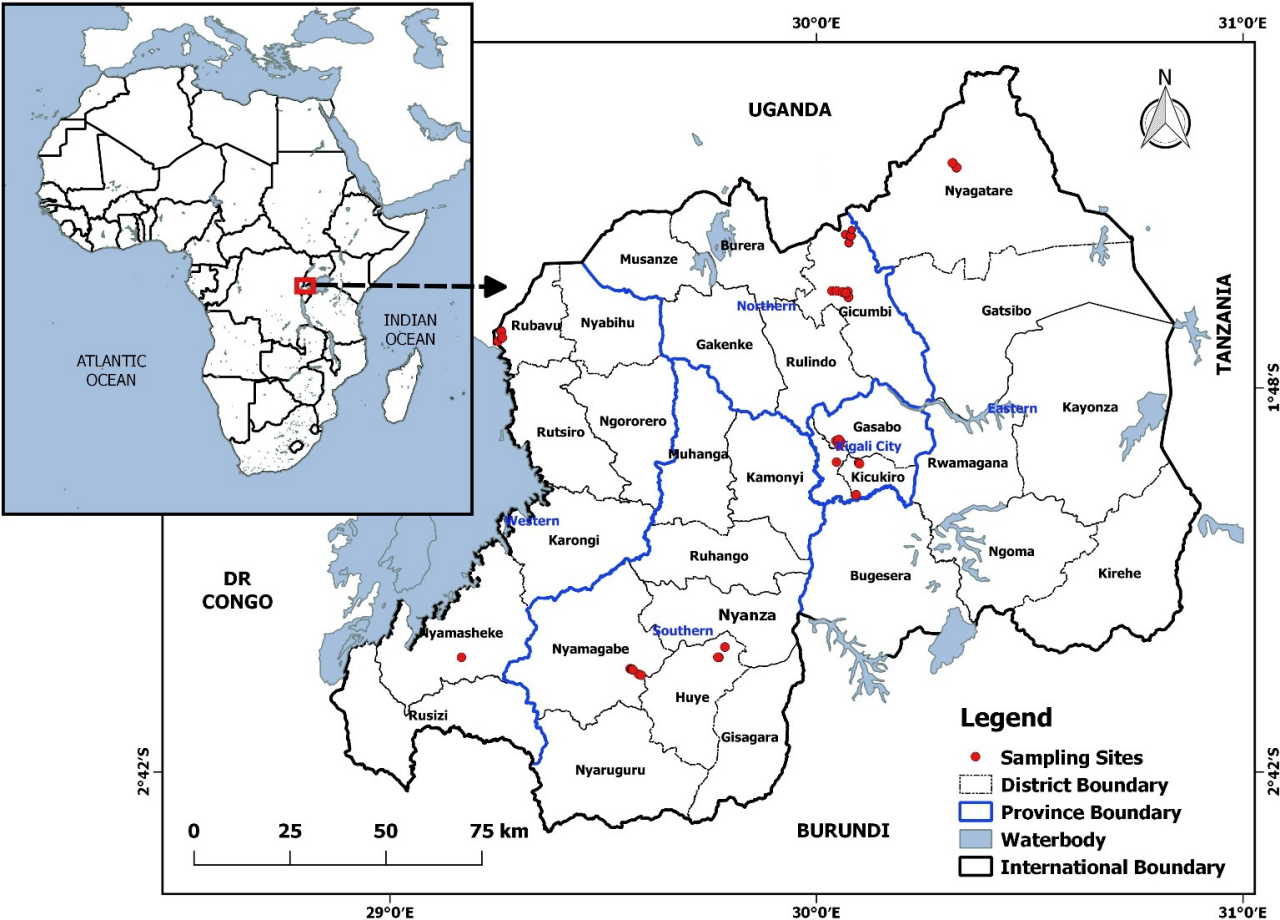
Map of sampling sites of chicken blood used in this study.

In total, 313 distinct IC, previously characterized morphologically (S1 Table) [27], were sampled from five agro-ecological zones [51, 52, 53, 55, and 102 were sampled from Central South (CS), North West (NW), Central North (CN), South West (SW), and East (E), respectively] (S1 Fig and Fig 1). Indigenous Chicken populations were reckoned according to agro-ecological zones [28]. Households having IC were randomly selected considering a minimum distance of 500 meters between them to ensure sampling of unrelated birds [29]. Twelve (12) exotic commercial chicken breeds (2 kuroilers, 5 Isa brown layers and 5 cobb broilers) were included as references. These exotic breeds have been developed from several parent breeds which are not usually divulged by breeder companies and, therefore, are marketed as commercial hybrids under trade names. They were genetically selected for performance traits associated with egg (layers), meat (broilers) or both egg and meat (kuroilers) production (S1 Table).

A single blood drop was drawn from veins in the wing of each bird and placed on Whatman FTA^™^ filter cards, left to dry in a cool place for approximately one hour, and held in reserve in discrete envelopes at room temperature awaiting further processing. The isolation of genomic DNA was done using Smith and Burgoyne’s boiling method [30]. The quality of genomic DNA was ascertained through gel electrophoresis using 1% agarose. A NanoDrop Spectrophotometer (Thermo Scientific ^TM^ Nanodrop 2000) was used to quantify the total DNA, which was adjusted to 10ng/μl before use in the subsequent steps of polymerase chain reaction (PCR) and genotyping.

### PCR amplification and DNA polymorphism

Twenty-eight fluorescently-labelled polymorphic SSR markers were chosen based on the extent of polymorphism shown by a high polymorphism information content and the genome coverage consistent across previous studies [31]. The PCR reactions had a total volume of 10μl consisting of 30ng target DNA, 5μl of One Taq 2MM and 0.2μl of each forward and reverse primer. The amplifications were done in a thermocycler (Applied Biosystems 9700 Thermal Cycler Gene Amp®) and entailed the first denaturation at 94°C for 3 minutes, 30 cycles of denaturation at 94ºC for 30 seconds, the primer annealing at temperatures ranging between 58°C and 64°C based on the primer components (Table 1) for 1 minute, and extension at 72°C for 2 minutes. The last extension step was done at 72°C for 10 minutes. The PCR products of different fluorescent tags were combined according to the exhibited colour and intensity of bands to create uniform signal strength. Hi-Di formimide was used to denature the combined amplicons at 95°C for 3 minutes, this step was followed by capillary electrophoresis separation in an ABI3730 DNA genetic analyzer by using GeneScan- 500 Internal LIZ and 1200 Internal LIZ Size Standards. The resultant fragment analysis data and sizes of alleles were counted using GENEMAPPER software v. 4.1 (Applied Biosystems).

**Table 1:** Sequences and physical information of 28 SSR markers used for PCR amplification.

Nam	Allele size (base-pairs)	Forward Primer 5'- 3'	Reverse primer 3'-5'	Annealing temperature (Tm: ^o^C)
ADL0268	102–116	CTCCACCCCTCTCAGAACTA	CAACTTCCCATCTACCTACT	60
MCW0206	221–249	ACATCTAGAATTGACTGTTCAC	CTTGACAGTGATGCATTAAATG	60
LEI0166	354–370	CTCCTGCCCTTAGCTACGCA	TATCCCCTGGCTGGGAGTTT	60
MCW0295	88–106	ATCACTACAGAACACCCTCTC	TATGTATGCACGCAGATATCC	60
MCW0081	112–135	GTTGCTGAGAGCCTGGTGCAG	CCTGTATGTGGAATTACTTCTC	60
MCW0014	164–182	TATTGGCTCTAGGAACTGTC	GAAATGAAGGTAAGACTAGC	58
MCW0183	296–326	ATCCCAGTGTCGAGTATCCGA	TGAGATTTACTGGAGCCTGCC	58
ADL0278	114–126	CCAGCAGTCTACCTTCCTAT	TGTCATCCAAGAACAGTGTG	60
MCW0067	176–186	GCACTACTGTGTGCTGCAGTTT	GAGATGTAGTTGCCACATTCCGAC	60
MCW0104	190–234	TAGCACAACTCAAGCTGTGAG	AGACTTGCACAGCTGTGTACC	60
MCW0123	76–100	CCACTAGAAAAGAACATCCTC	GGCTGATGTAAGAAGGGATGA	60
MCW0330	256–300	TGGACCTCATCAGTCTGACAG	AATGTTCTCATAGAGTTCCTGC	60
MCW0165	114–118	CAGACATGCATGCCCAGATGA	GATCCAGTCCTGCAGGCTGC	60
MCW0069	158–176	GCACTCGAGAAAACTTCCTGCG	ATTGCTTCAGCAAGCATGGGAGGA	60
MCW0248	205–225	GTTGTTCAAAAGAAGATGCATG	TTGCATTAACTGGGCACTTTC	60
MCW0111	96–120	GCTCCATGTGAAGTGGTTTA	ATGTCCACTTGTCAATGATG	60
MCW0020	179–185	TCTTCTTTGACATGAATTGGCA	GCAAGGAAGATTTTGTACAAAATC	60
MCW0034	212–246	TGCACGCACTTACATACTTAGAGA	TGTCCTTCCAATTACATTCATGGG	60
LEI0234	216–364	ATGCATCAGATTGGTATTCAA	CGTGGCTGTGAACAAATATG	60
MCW0103	266–270	AACTGCGTTGAGAGTGAATGC	TTTCCTAACTGGATGCTTCTG	64
MCW0222	220–226	GCAGTTACATTGAAATGATTCC	TTCTCAAAACACCTAGAAGAC	60
MCW0016	162–206	ATGGCGCAGAAGGCAAAGCGATAT	TGGCTTCTGAAGCAGTTGCTATGG	60
MCW0037	154–160	ACCGGTGCCATCAATTACCTATTA	GAAAGCTCACATGACACTGCGAAA	64
MCW0098	261–265	GGCTGCTTTGTGCTCTTCTCG	CGATGGTCGTAATTCTCACGT	60
LEI0094	247–287	GATCTCACCAGTATGAGCTGC	TCTCACACTGTAACACAGTGC	60
MCW0284	235–243	GCCTTAGGAAAAACTCCTAAGG	CAGAGCTGGATTGGTGTCAAG	60
MCW0078	135–147	CCACACGGAGAGGAGAAGGTCT	TAGCATATGAGTGTACTGAGCTTC	60
LEI0192	244–370	TGCCAGAGCTTCAGTCTGT	GTCATTACTGTTATGTTTATTGC	60
ADL0112	120–134	GGCTTAAGCTGACCCATTAT	ATCTCAAATGTAATGCGTGC	58
MCW0216	139–149	GGGTTTTACAGGATGGGACG	AGTTTCACTCCCAGGGCTCG	60

Source: FAO [32]

### Statistical analysis

#### Genetic diversity and relationship

The polymorphism information content (PIC) was estimated using Powermarker v.3.25 [6]. GenAlEx v.6.5 was used to estimate the allele frequencies, total alleles, expected heterozygosity (He), observed heterozygosity (Ho), and Wright’s F-statistics as well as other parameters such as inbreeding coefficient over all populations (Fis), among populations (Fit) and within populations (Fst) for 28 microsatellite markers [7]. Jackknifing across populations using FSTAT v.2.9.4 produced standard deviation values that were used to obtain tests of significance per microsatellite locus by creating confidence intervals at 95% and 99% [8].

GENETIX v.4.05.2 was used to estimate genetic variation per breed (He, Ho) and the average number of alleles [9]. Gene flow [10] was calculated using Powermarker v.3.25 [6]. Pairwise Fst values, which are indications of the fraction of genetic variation attributed to population sub-structuring, were calculated for various population pairs using GenAlEx v.6.5 [7]. Analysis of molecular variance (AMOVA) was computed using GenAlEx v.6.5 for within and among pre-grouped populations [7]. Powermarker v 3.25 was used to assess genotype frequencies for nonconformity with Hardy-Weinberg equilibrium (HWE) in addition to linkage disequilibrium by performing Pearson's chi-squared test (χ)^2^ [6]. GenAlEx v.6.5 [7] was used to approximate Nei’s standard genetic distances [11] among population pairs. The Neighbour-Joining (NJ) programme was used to develop an unrooted NJ cladogram using the Darwin software v.6.0 according to pairwise kinship distance matrix between populations [12]. A consensus tree assessed by 1,000 bootstraps all through the group of loci was created.

#### Population structure

The possible sum of clusters was approximated using the Evanno method [13] as reported by Dent Earl and Bridgett [14]. A set of rules applied in STRUCTURE v.2.3.4 was used to group entities based on multi-locus genotypes [15]. The evaluation entailed an admixture model alongside interrelated allele frequencies. During the STRUCTURE analysis, 5 replications of K (presumed sum of subpopulations), extending from 1 to 20 were used together with 100,000 reiterations of Markov Chain Monte Carlo (MCMC) and 50,000 burn-in period in the admixture model. Each estimation of K was redone 5 times to ensure the reproducibility of the outcomes. CLUMPAK (CLUMPAK server), which is a tool used to single out clustering types and bundle population structure deductions across K was used. The Factorial Correspondence Analysis (FCA), which is a multivariate model of analysis, was conducted to observe the associations between entities from unlike zones and to evaluate probable admixtures between the populations. The main variables were the frequencies of alleles at all loci in the populations. The FCA was computed using GENETIX v.4.05.2 [9].

### Results

#### Genetic diversity

##### Marker polymorphism across the studied IC populations

The parameters of the variability of the investigated loci are shown in Table 2. Overall, 305 alleles were observed at the 28 microsatellite loci with an average of 10.89 alleles per microsatellite marker. The total sum of alleles ranged from 2 (MCW0037) to 22 (LEI0192). The effective number of alleles (NE) ranged between 1.6504 (MCW0078) and 8.901 (LEI0234), with an overall mean of 3.8194. The PIC ranged from 0.3488 (MCW0103) to 0.8775 (LEI0234). Out of the total number of alleles, 20% were private alleles (60), whereas ADL0112 revealed the maximum sum of private alleles (6). The within-population insufficiency in heterozygosity (as determined by F_IS_ factor), extended between −1.00 (MCW0037) and 0.338 (LEI0234) with a mean of 0.041 for all loci. The inbreeding coefficient among populations (F_IT_) values ranged from -1.00 (MCW0037) to 0.354 (LEI0234), with a mean of 0.089. Global population differentiation (evaluated by F_ST_) was estimated at 0.054. The contribution of 28 microsatellites for population segregation (determined by F_ST_ statistics) varied from 0.000 (MCW0037) to 0.158 (ADL0268). The overall F-statistics differed significantly (p<0.05) from zero. This differentiation had a significant contribution from all loci. The values for Ho ranged from 0.3015 (MCW0165) to 1 (MCW0037), with an overall mean of 0.6155, while the values of He ranged from 0.394 (MCW0078) to 0.8877 (LEI0234), with a general mean of 0.688. The average number of migrants per generation (Nm) in the whole population and across all the loci was found to be 6.06. Only 10% of the loci in all IC populations, did not differ considerably (p >0.05) from the HWE.

**Table 2:** Marker polymorphism and diversity parameters across studied IC populations in Rwanda.

Loci	MAF	NG	NA	NE	NPA	He	Ho	PIC	I	Fis	Fit	Fst	Nm	HWE pV
ADL0112	0.499	27	16	2.720	6	0.632	0.594	0.572	1.318	0.097	0.128	0.034	7.006	0.000
ADL0268	0.245	39	14	6.241	3	0.840	0.582	0.820	2.022	0.176	0.306	0.158	1.332	0.000
ADL0278	0.300	39	12	5.349	4	0.813	0.548	0.789	1.885	0.252	0.283	0.041	5.869	0.000
LEI0094	0.392	45	17	4.360	3	0.771	0.714	0.744	1.867	0.017	0.034	0.017	14.344	0.000
LEI0192	0.317	66	22	5.699	4	0.825	0.775	0.806	2.149	-0.005	0.036	0.041	5.829	0.000
LEI0234	0.177	77	17	8.902	2	0.888	0.569	0.878	2.393	0.338	0.354	0.024	10.202	0.000
MCW0014	0.512	29	10	3.107	1	0.678	0.486	0.645	1.493	0.142	0.263	0.142	1.517	0.000
MCW0016	0.317	39	15	4.699	4	0.787	0.772	0.759	1.841	0.002	0.023	0.021	11.392	0.000
MCW0020	0.305	29	8	4.661	0	0.785	0.720	0.753	1.676	0.050	0.095	0.047	5.027	0.000
MCW0034	0.351	46	14	5.211	5	0.808	0.775	0.788	1.927	-0.003	0.032	0.035	6.965	0.191
MCW0037	0.500	1	2	2.000	0	0.500	1.000	0.375	0.693	-1.000	-1.000	0.000		0.000
MCW0067	0.395	31	11	3.573	1	0.720	0.680	0.679	1.622	0.038	0.137	0.103	2.181	0.000
MCW0069	0.339	26	10	3.671	0	0.728	0.739	0.680	1.503	-0.011	0.028	0.038	6.309	0.104
MCW0078	0.766	11	5	1.650	0	0.394	0.369	0.372	0.820	-0.006	0.006	0.011	21.491	0.015
MCW0081	0.494	42	11	3.001	1	0.667	0.560	0.622	1.483	0.126	0.156	0.034	7.140	0.000
MCW0098	0.465	27	9	2.571	1	0.611	0.523	0.535	1.176	0.105	0.170	0.072	3.212	0.000
MCW0103	0.708	9	6	1.736	2	0.424	0.375	0.349	0.693	0.131	0.160	0.033	7.343	0.000
MCW0104	0.489	43	18	3.271	4	0.694	0.649	0.662	1.701	0.066	0.096	0.033	7.385	0.000
MCW0111	0.595	21	8	2.440	0	0.590	0.483	0.550	1.226	0.110	0.141	0.035	6.800	0.000
MCW0123	0.523	38	14	3.103	3	0.678	0.640	0.650	1.568	0.015	0.031	0.016	15.002	0.000
MCW0165	0.635	7	4	1.924	0	0.480	0.302	0.386	0.755	0.325	0.341	0.024	10.050	0.000
MCW0183	0.292	34	11	5.516	3	0.819	0.659	0.796	1.873	0.119	0.189	0.080	2.885	0.000
MCW0206	0.394	24	9	3.992	2	0.750	0.699	0.714	1.583	-0.004	0.044	0.048	5.000	0.000
MCW0222	0.400	11	6	2.972	2	0.664	0.646	0.600	1.210	-0.030	0.023	0.051	4.641	0.000
MCW0248	0.679	6	4	1.816	1	0.449	0.492	0.366	0.713	-0.236	-0.185	0.041	5.864	0.344
MCW0284	0.368	29	8	3.900	0	0.744	0.689	0.706	1.620	0.050	0.117	0.070	3.321	0.000
MCW0295	0.465	34	13	3.482	3	0.713	0.579	0.680	1.632	0.131	0.214	0.096	2.341	0.000
MCW0330	0.302	26	11	5.376	5	0.814	0.615	0.790	1.827	0.147	0.281	0.157	1.339	0.000
Mean	0.437	30.571	10.893	3.819	2.140	0.688	0.616	0.645	1.510	0.041	0.089	0.054	6.060	
Total			305		60									

MAF, major allele frequency; NG, number of genotypes; NA, number of alleles; NPA, number of private allele; Ne, number of effective alleles; I, Shannon's information index; He, expected heterozygosity; Ho, observed heterozygosity; PIC, polymorphic information content, Nm: number of migrants, F, inbreeding coefficient over all populations (F_IS_), among populations (F_IT_) and within populations (F_ST_), HWE pV, Hardy-Weinberg equilibrium p-value based on chi square test (There is a deviation from HWE at p<0.05)

##### Genetic diversity indices for IC populations from each agro-ecological zone

Genetic diversity indices for IC from each zone is summarized in Table 3. All the loci were polymorphic. The observed and expected frequencies of heterozygote were not statistically different (p>0.05), hence, the inbreeding coefficient (F) estimates observed were not substantially different from zero. The mean sum of alleles varied from 5.143 to 8.25. The highest count of alleles (8.2) was found in the Eastern IC population. The highest count of private alleles (21) was observed in the Eastern population, while the NW population did not harbor any private allele. The effective sum of alleles ranged from 3.311 to 3.62. The Shannon Index (I), which is an expression of population diversity in a particular habitat, was high in the SW (1.458) and low in exotic chicken (1.305). Furthermore, the lowest observed heterozygosity was in the CS (0.598) while the highest was recorded in exotic chicken population (0.667). The expected heterozygosity in the populations ranged from0.644 (CN) to 0.680 (SW).

**Table 3:** Common genetic diversity indices as revealed among IC populations in Rwanda.

Populations	N	%PL	NA	PA	Ne	Ho	He	uHe	F	I
Central North	51	100	6.929	6	3.354	0.623	0.644	0.650	0.021	1.322
Central South	55	100	7.286	15	3.359	0.598	0.661	0.668	0.077	1.372
Exotic chicken	12	100	5.143	4	3.386	0.667	0.665	0.669	-0.019	1.305
East	102	100	8.250	21	3.367	0.611	0.654	0.657	0.056	1.358
North West	52	100	6.500	0	3.311	0.613	0.645	0.651	0.042	1.306
South West	53	100	7.964	14	3.620	0.626	0.680	0.686	0.063	1.458
Total	325	100	7.011	60	3.400	0.623	0.658	0.668	0.040	1.353

N, Number of chickens, % PL, Proportion of polymorphic loci, NA, number of alleles; PA, number of private allele; Ne, number of effective alleles He, expected heterozygosity Ho, observed heterozygosity uHe: unbiased expected heterozygosity F, inbreeding coefficient I, Shannon's information index.

The p-values of HWE are summarized in Table 4 and confirm that Ho and He did not differ significantly (P>0.05). Thus, taking all the loci into account none of the IC populations diverged from the HWE law.

**Table 4:** Tests for the Hardy-Weinberg equilibrium probability of loci in the IC population in Rwanda.

Locus	North West	Central North	Central South	East	North-south	Exotic chicken
ADL0112	0.551	0.000	0.000	0.003	0.000	0.028
ADL0268	0.000	0.000	0.163	0.000	0.000	0.330
ADL0278	0.000	0.000	0.000	0.000	0.003	0.349
LEI0094	0.001	0.976	0.000	0.051	0.001	0.812
LEI0192	0.000	0.000	0.000	0.002	0.024	0.913
LEI0234	0.000	0.000	0.000	0.099	0.000	0.720
MCW0014	0.000	0.000	0.000	0.000	0.000	0.634
MCW0016	0.012	0.000	0.000	0.239	0.108	0.200
MCW0020	0.048	0.586	0.190	0.620	0.000	0.980
MCW0034	0.050	0.735	0.316	0.000	0.816	0.412
MCW0037	0.000	0.000	0.000	0.000	0.000	0.001
MCW0067	0.000	0.000	0.870	0.000	0.000	0.095
MCW0069	0.965	0.529	0.971	0.967	0.295	0.279
MCW0078	0.911	0.251	0.985	0.232	0.003	0.916
MCW0081	0.739	0.000	0.000	0.000	0.000	0.004
MCW0098	0.681	0.000	0.000	0.000	0.000	0.005
MCW0103	0.012	0.752	0.000	0.913	0.000	0.574
MCW0104	0.001	1.000	0.355	0.000	0.755	0.213
MCW0111	0.046	0.189	0.127	0.003	0.687	0.545
MCW0123	0.503	0.909	0.000	0.002	0.000	0.003
MCW0165	0.540	0.000	0.004	0.000	0.018	0.327
MCW0183	0.000	0.010	0.000	0.000	0.012	0.001
MCW0206	0.590	0.020	0.009	0.908	0.000	0.658
MCW0222	0.000	0.096	0.000	0.783	0.968	0.283
MCW0248	0.429	0.922	0.057	0.991	0.247	0.035
MCW0284	0.121	0.021	0.846	0.000	0.000	0.437
MCW0295	0.279	0.000	0.017	0.000	0.046	0.015
MCW0330	0.633	0.992	0.000	0.000	0.150	0.001

P-values <0.05 show the genotype frequencies for nonconformity with Hardy-Weinberg Equilibrium (HWE) based on Chi square test

Analysis of molecular variance revealed that ninety-two percent (92%) of the total variation originated from variation within populations (Table 5).

**Table 5:** Analysis of molecular variance of all loci for the IC population in Rwanda.

Source	Degree of freedom	Sum square	Mean square	Estimated variances	% of estimated variances
**Among Populations**	5	574.201	114.840	1.838	8%
**Within Populations**	319	6346.643	19.895	19.895	92%
**Total**	324	6920.843		21.733	100%

#### Genetic relationship

**Fig 2:**
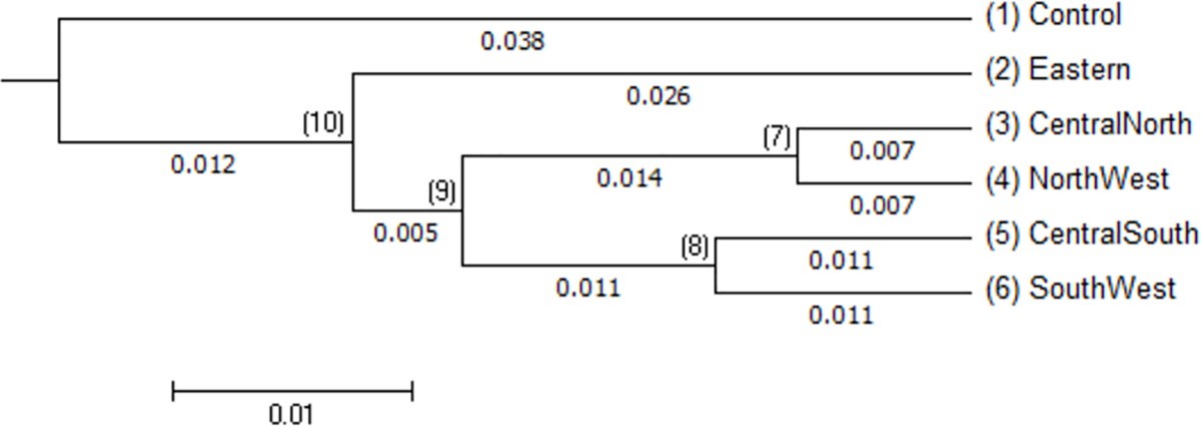
Neighbour-Joining pair-wise of the IC population in Rwanda.

The matrix of pairwise genetic distances between populations (Table 6 and Fig 2) showed low genetic distance (0.029) between NW and CN populations. A similar trend was observed in SW and CS (0.048). On the other hand, by considering only the IC populations, the highest genetic distance was observed between E and SW populations (0.125). The genetic distance between the IC population in Rwanda and exotic chicken was relatively high (0.231).

**Table 6:** Genetic distance among the IC population in Rwanda.

Populations	North West	Central North	Central South	Exotic chicken	East
Central North	0.029				
Central South	0.094	0.077			
Exotic chicken	0.199	0.213	0.231		
East	0.112	0.097	0.117	0.196	
South West	0.104	0.092	0.048	0.118	0.125

The extent of genetic distinction among the population with regard to allele frequencies (F_ST_) and gene flow (Nm) are presented in Table 7. The results revealed a low genetic differentiation and a high gene flow between CN and NW, and likewise between SW and CS. A relatively high gene differentiation, however, was found between the E population and other populations.

**Table 7:** Gene flow (upper diagonal) and Gene differentiation (lower diagonal).

Populations	Central North	Central South	Exotic chicken	East	North West	South West
Central North		2.304	1.412	2.051	6.274	2.040
Central South	0.022		0.925	1.471	1.533	3.847
Exotic chicken	0.052	0.058		3.432	1.188	2.791
East	0.025	0.027	0.050		1.783	1.560
North West	0.012	0.026	0.053	0.028		1.471
South West	0.026	0.014	0.036	0.028	0.027	

**Fig 3:**
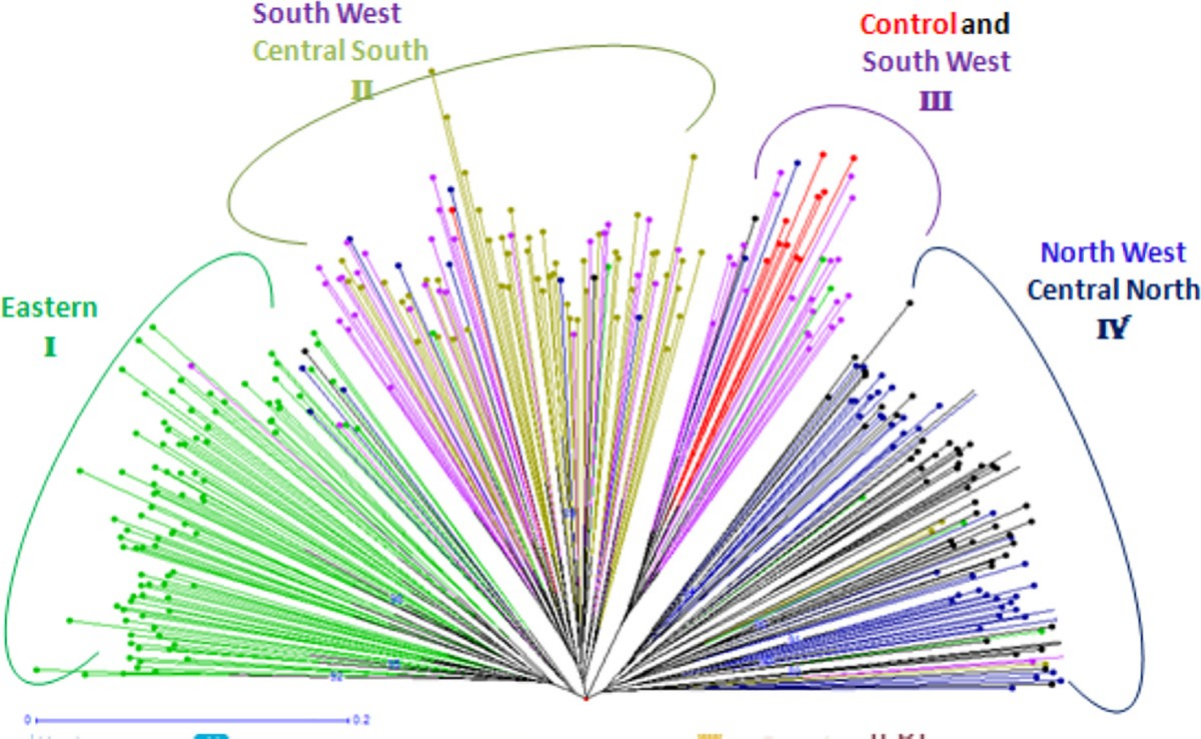
Neighbour-Joining tree of the clustering pattern among IC populations in Rwanda.

The phylogenetic relationship by the Neighbour-Joining tree showed four (4) IC genetic clusters, namely I, II, III and IV (Fig 3). The eastern population stands alone unlike the other populations: IC populations from the NW clustered together with those from the CN. Few individuals from the SW population clustered together with the exotic chicken in group III, and finally the rest of SW individuals clustered with those from the CS in group II (Fig 3).

### Population structure

**Fig 4:**
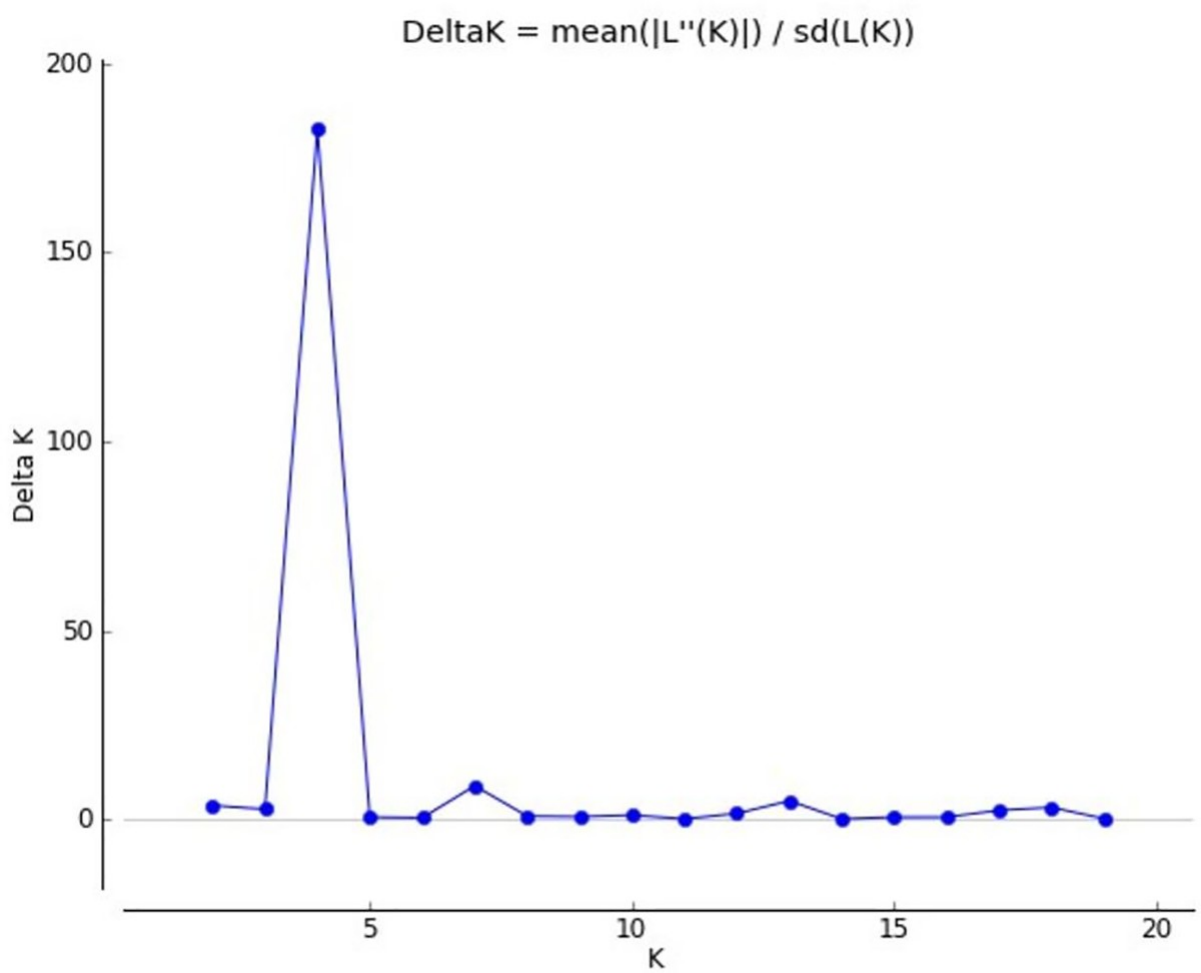
Delta K (ΔK) approximating the more possible number of clusters in IC populations in Rwanda.

Data from the Bayesian cluster analysis showed the existence of four (4) main gene pools in the whole IC population in Rwanda. The highest value for *ΔK* was obtained for K = 4 (Table 8 and Fig 4). The first gene pool (I) was composed of CN and NW populations. The second gene pool (II) was made of the Eastern population only. The third (III) included individual from SW and CS and the fourth gene pool (IV) was composed of the remaining individuals of SW and exotic chicken. A high proportion of the admixture was observed in the gene pool III.

**Table 8:** Number of clusters (K) based on the progression of the average estimate of Ln likelihood of data in IC populations in Rwanda.

K	Replication	Mean LnP(K)	Stdev LnP(K)	Ln’(K)	ILn”(K)I	Delta K
1	5	-27680.120000	0.192354	-	-	-
2	5	-26645.700000	81.765916	1034.420000	301.520000	3.687600
3	5	-25912.800000	30.968694	732.900000	82.920000	2.677543
**4**	**5**	**-25262.820000**	**3.056469**	**649.980000**	**558.300000**	**182.661785**
5	5	-25171.140000	37.017671	91.680000	21.920000	0.592150
6	5	-25057.540000	46.761341	113.600000	19.200000	0.410596
7	5	-24963.140000	9.161496	94.400000	81.200000	8.863182
8	5	-24949.940000	63.605566	13.200000	55.340000	0.870050
9	5	-24881.400000	42.680968	68.540000	29.880000	0.700078
10	5	-24842.740000	77.738491	38.660000	87.640000	1.127369
11	5	-24891.720000	114.353824	-48.980000	14.060000	0.122952
12	5	-24954.760000	210.975195	-63.040000	330.240000	1.565302
13	5	-24687.560000	104.370245	267.200000	510.500000	4.891241
14	5	-24930.860000	402.389690	-243.300000	41.440000	0.102985
15	5	-25132.720000	914.525050	-201.860000	542.960000	0.593707
16	5	-24791.620000	296.572178	341.100000	183.320000	0.618129
17	5	-24633.840000	54.568333	157.780000	129.560000	2.374271
18	5	-24605.620000	64.775126	28.220000	204.760000	3.161090
19	5	-24782.160000	498.369745	-176.540000	100.700000	0.202059
20	5	-24858.000000	559.214181	-75.840000	-	-

**Fig 5:**
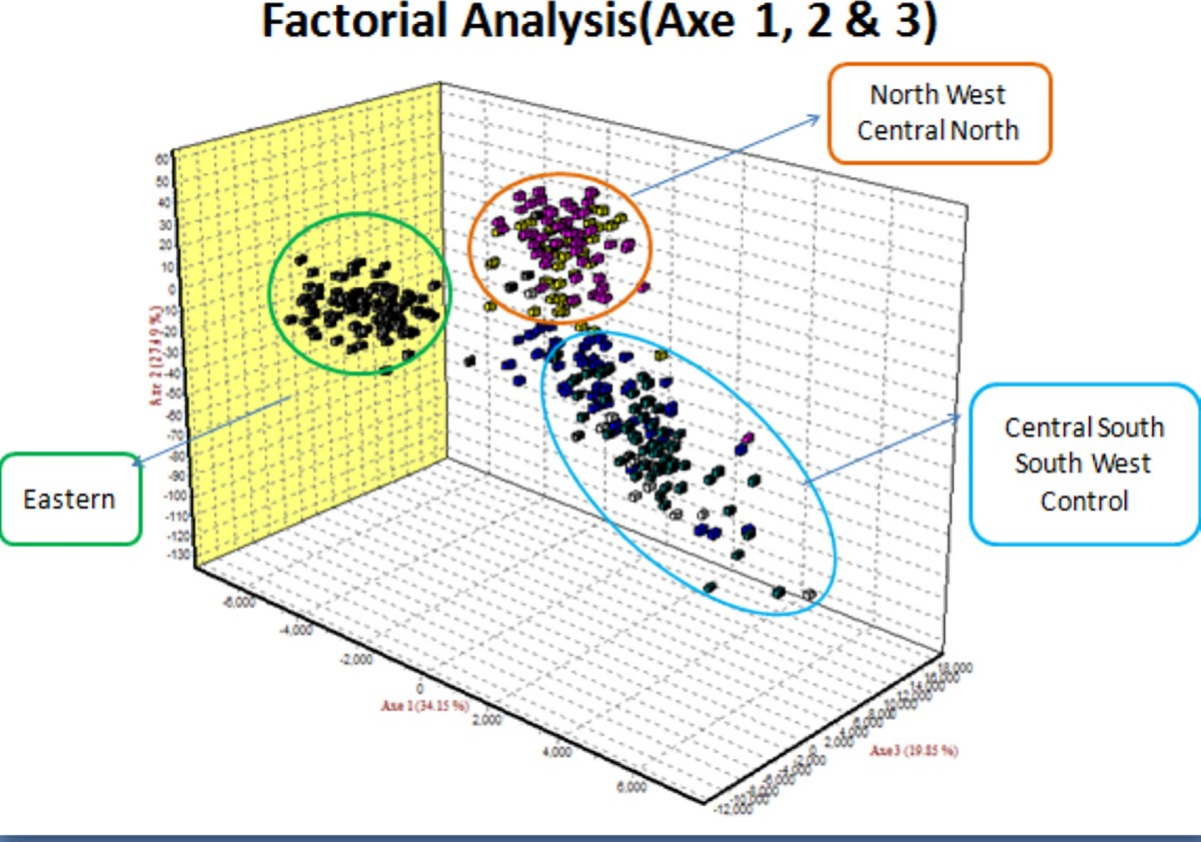
Factorial correspondence analysis.

The results of the Factorial Correspondence Analysis (FCA) are depicted in Fig 5. It showed tree clusters whereby the Eastern region was still standing alone. NW and CN populations clustered together. Finally, the majority of individuals from the CS, SW and exotic chicken were in the same group.

## Discussion

### Genetic diversity

The average PIC was the best index to estimate the polymorphism of alleles [16]. It showed that more information could be obtained from the loci when PIC>0.5. On the other hand, 0.25<PIC<0.5 was an indication of a moderately instructive locus, whereas PIC<0.25 indicated a vaguely informative locus [33]. In this study, 82.3% of all loci were highly informative, which confirmed that they were suitable for estimating the genetic diversity of IC populations in Rwanda. The highest value of PIC (0.87) was that of LEI0234 and the mean PIC was 0.6451. The PIC values found in this study exceeded those (0.29–080) of Cameroon’s IC [17], and (0.31–0.49) of Chinese IC [7,8], but lower than those obtained by Tang for black-bone IC breeds (0.67) [34]. The mean frequency of alleles per marker found in this study (10.89) exceeded those recorded in previous reports in Cameroon (9.04) [17], Ghana (7.8) [35], Iran (5.4) [36], China (3.8) [37], Egypt (7.3) [38], Pakistan (9.1) [39] and Vietnam (6.41) [40]. The values obtained in this study were, however, lower than those from Brazilian (13.3) [41] and were in the same range as those from Ethiopian chicken ecotypes (10.6) [42].

The mean number of effective alleles (3.81) obtained was higher than 3.13 observed in Cameroon [17] and Indian chicken [21]. Heterozygosity can also be considered in genetic diversity. The degree of mean population heterozygosity is an indication of the level of population constancy. Low population heterozygosity informs high population genetic constancy [43]. The present study indicated that Ho of the different IC population varied from 0.3015 to 1 with an overall mean value of 0.6155, while He ranged from 0.394 to 0.887 with an overall average of 0.688.

This study also discovered that the values of Ho and He were similar. As a result, there was no significant difference between zero and the resultant F estimates (0.040), which suggested that the IC populations were in HWE. An implication of this supposition is that the population is under artificial selection, which is indicative of population stability. However, the little variation observed between Ho and He could be attributed to discrepancies in sample size, location, population composition, and the origin of microsatellite markers [44].

The IC populations in Rwanda had a similar level of diversity as their Ethiopian [45], Egyptian [38] and Cameroonian [8] counterparts, but had lower and higher diversity than those observed in southern China [19], European and Asian IC breeds [35], respectively. Among Rwanda IC, all populations showed a significantly high degree of inbreeding, which could have an impact on trait fixation in the populations. This degree of inbreeding exceeded that observed for Yunnan IC breeds (0.25) [8] and Turkish IC (0.301) depicted with 10 SSR loci [44]. The F_ST_ value (0.054) revealing the diversity between IC populations in Rwanda was higher than 0.048 for Ethiopian IC ecotypes [46] and (0.003–0.040) for Kenyan IC [47] and lower than 0.080 found in Cameroonian IC [17].

### Genetic relationships

Wright’s F-statistics showing the inbreeding coefficient in this study was 0.041, which was higher than 0.03 found in Cameroon [17], but was similar to values obtained in many Chinese IC [18]. The F_ST_ permits the approximation of migratory entities in a population per generation (Nm) based on loci. In IC populations in Rwanda, Nm varied from 1.332 to 21.491, with an average of 6.060. This value was higher than that obtained in Cameroun [17].

The number of private alleles (PA) distributed all through the ecotypes showed that there was high genetic diversity between populations. In this study, the number of PA was higher in the East (21) followed by CS (15) and SW (14). The NW population, however, did not exhibit any private allele (0). Despite, the number of private alleles being a good indicator of population relationship and structure, further studies need to be carried out to identify possible traits that may be controlled by these private alleles. The total number of private alleles in this study (60) was higher than that (24) found in Cameroun [17].

Findings from AMOVA showed that the largest portion of the genetic variation in IC populations in Rwanda existed in individuals within the population (92%). A comparable trend was noted in the Tanzanian [48], Ethiopian [17] and Cameroonian [17] IC ecotypes. The quality of the product, cultural uses of chicken, and the ease with which chicken adapts to the environment are the factors that motivate small-scale farmers to rear IC. These factors highlight the importance of within-population diversity as a key incentive in rearing IC [49].

Genetic distance within a population is a useful indicator of separation between various sub-populations. The key assumption of Nei's standard genetic distance is that hereditary dissimilarities are caused by mutations and genetic drift, whereas Reynolds distance assumes that the increase of genetic differences is due to genetic drift only [11]. The genetic distance between IC populations in SW and CS as well as between NW and CN were not significantly different (P>0.05). It was noted that these regions border each other, thereby implying that there is a high likelihood of sharing genetic materials. Another possible explanation is that these regions could be highly favorable to the IC population or IC populations in these regions could be big enough to prevent mutation and genetic drift. The genetic distances reported in this study fluctuated from 0.029 to 0.213. These values are in the range of those found in Egyptian IC [38] and in Chinese IC populations [50]. They are, however, higher than those observed in Chinese Bian chicken [19].

When estimating genetic differentiation using allele frequency in such scenarios, the genetic variance between populations can be explained by four major forces, namely, selection, mutation, migration, and genetic drift [44]. Even though mutation plays a critical role in the long term, short-term evolution is mainly influenced by genetic drift in cases where populations segregated by reproduction [51]. Genetic distance analysis is used to show how close two populations are in relation to each other. The smaller the distance, the closer the two populations are to one another and vice versa [11]. IC populations showed segregation by distance and appeared to be at equipoise under the influence of dispersal and genetic drift. There is a high likelihood that these chickens were present at their current locations earlier than it had been assumed because there was not enough time for segregation due to distance to have come into play. Furthermore, long-distance gene dispersion is not satisfactorily evident to deter genetic deviation. For this, further investigations need to be conducted using more markers, for example, high-density SNP arrays and mitochondrial DNA which was also conducted concurrently with the current study.

### Phylogenetic relationship and population structure

The genetic similarity in a collection of breeds with high diversity can be resolved efficiently by cluster analysis, which facilitates the identification of individuals with similar or diverse multi-locus genotypes [52]. A number of IC populations clustered together indicates genetic affinities between them [53]. In our study, the cluster based on the neighbour-joining approach revealed grouping arrays of association and genetic relationships among individuals. These individuals were grouped into four clusters formed by ecotypes from distinct collection sites (NW and CN; SW1 and CS; SW2 with exotic chicken and East alone). This close genetic relationship may indicate a common genetic background [54]. A cluster shows the degree of inbreeding and populations that could be sharing the identical ancestral lineage [55]. There is also similarities in morphological characteristics between the IC populations clustered together [56]. This was confirmed by the structure analysis which revealed four gene pools across IC in Rwanda. These gene pools are distributed exactly according to the different clusters as shown by the neighbour-joining method. The observed gene pools could be accounted for by the sum of private alleles recorded in the population besides the genetic distance between populations. For example, the Eastern region recorded the highest frequency of private alleles, whereas the NW had the lowest number. This observation could be attributed to the large population size of IC in the Eastern region out of all the study sites, which minimized gene inflow in this area. Conversely, the lowest number of IC was noted in the NW region, which could be interpreted to mean that the majority of chicken keepers in this area either buy chicken or exchange cocks from the neighbouring areas such as CN. Consequently, there is a high influx of genes in these regions. This is not surprising since these areas border each other geographically. These findings corroborated the observations of a study conducted in Kenya where the Mantel test had uncovered a positive association between hereditary and geographic distances [57]. Our study also confirmed that geographic distances affected the population’s genetic structure [57]. The portion of SW chicken populations that clustered with the exotic chicken could be attributed to the fact that different crossing programmes between IC and improved chicken breeds have been introduced in that region to improve the genetic potential of IC in Rwanda [58].

## Conclusion

The results from this study are the first to recount the genetic diversity and constitution of IC from Rwanda. Overall, the IC populations in Rwanda had high levels of significant genetic variability as per different genetic diversity parameters applied in this study. Therefore, data on genetic diversity estimated by assimilating within and between population variances may inform preservation strategies and the better establishment of priorities. In addition, this study found that IC in Rwanda belongs to four major gene pools that could be preserved independently to uphold their genetic diversity. Generally, these findings provide the fundamental step in the direction of judicious decision-making before the development of genetic enhancement and preservation programmes without interfering with the uniqueness of IC in Rwanda.

## Supporting information

S1 Table Characteristics of indigenous and exotic chickens used in the study. (DOCX)

S1 Fig Agro ecological zones in Rwanda. (PDF)

## References

[1] NISR. Statistical Yearbook 2014 [Internet]. National Institute of Statistics of Rwanda 2014 10.1017/CBO9781107415324.004

[2] Mbuza F, Denis M, Janvier M, Xavier R. Characterization of low cost village Poultry production in Rwanda. Int J Livest Prod. Academic Journals; 2016;7: 76–82. 10.5897/IJLP2016.0300

[3] Mahoro J, Muasya TK, Mbuza F, Habimana R, Kahi AK. Characterization of indigenous chicken production systems in Rwanda. Poult Sci. 2017;96: 4245–4252. 10.3382/ps/pex240 29053822

[4] Mahoro J, Muasya TK, Mbuza F, Mbuthia J, Kahi AK. Farmers’ breeding practices and traits of economic importance for indigenous chicken in RWANDA. Trop Anim Health Prod. 2017;50: 121–128. 10.1007/s11250-017-1411-8 28948472

[5] MINAGRI. Rwanda Ministry of Agriculture & Animal Resources Annual Report FY 2010/2011. Annu Rep. 2011; Available: http://www.minagri.gov.rw/fileadmin/user_upload/documents/AnnualReports/Annual_Report_FY_2010_2011.pdf

[6] Liu K, Muse S V. PowerMaker: An integrated analysis environment for genetic maker analysis. Bioinformatics. 2005;21: 2128–2129. 10.1093/bioinformatics/bti282 15705655

[7] Peakall R, Smouse PE. GenAlEx 6.5: genetic analysis in Excel. Population genetic software for teaching and research—an update. Bioinformatics. Oxford University Press; 2012;28: 2537–9. 10.1093/bioinformatics/bts460 22820204 PMC3463245

[8] Goudet J. Computer Note. J Hered. 1995;86: 485–486. 10.1093/jhered/est020 23576670 PMC3678297

[9] Belkhir K, Borsa P, Chikhi L, Raufaste N, Bonhomme F. GENETIX 4.05, logiciel sous Windows TM pour la génétique des populations. Lab génome, Popul Interact CNRS Umr. 1996;5000: 1996–2004. Laboratoire, Génome, Populations, Interactions, CNRS UMR 5000, Université de Montpellier II, Montpellier, France.

[10] Wright. Evolution and the Genetics of Populations. 4: Univ Chicago Press Chicago 1978;Vol.IV: 157.

[11] Nei M. Genetic Distance between Populations. Am Nat. University of Chicago Press; 1972;106: 283–292. 10.1086/282771

[12] Perrier, X. and Jacquemoud-Collet JP. DARwin Software-CIRAD [Internet]. 2006 [cited 12 Oct 2018]. Available: http://darwin.cirad.fr/darwin

[13] Evanno G, Regnaut S, Goudet J. Detecting the number of clusters of individuals using the software structure: a simulation study. Mol Ecol. 2005;14: 2611–2620. 10.1111/j.1365-294X.2005.02553.x 15969739

[14] Earl DA, VonHoldt BM. STRUCTURE HARVESTER: A website and program for visualizing STRUCTURE output and implementing the Evanno method. Conserv Genet Resour. Springer Netherlands; 2012;4: 359–361. 10.1007/s12686-011-9548-7

[15] Falush D, Stephens M, Pritchard JK. Inference of population structure using multilocus genotype data: Dominant markers and null alleles. Mol Ecol Notes. 2007;7: 574–578. 10.1111/j.1471-8286.2007.01758.x 18784791 PMC1974779

[16] Kaya M, Yildiz MA. Genetic diversity among Turkish native chickens, Denizli and Gerze, estimated by microsatellite markers. Biochem Genet. 2008; 10.1007/s10528-008-9164-8 18500550 PMC2480610

[17] Keambou TC, Hako BA, Ommeh S, Bembide C, Ngono EP, Manjeli Y, et al Genetic diversity of the cameroon indigenous chicken ecotypes. Int J Poult Sci. 2014;13: 279–291. doi=ijps.2014.279.291&linkid=pdf

[18] Huo J, Wu G, Chen T, Huo H, Yuan F, Liu L, et al Genetic diversity of local Yunnan chicken breeds and their relationships with Red Junglefowl. Genet Mol Res Mol Res. 2014;13: 3371–3383. 10.4238/2014.April.29.16 24841782

[19] Ding FX, Zhang GX, Wang JY, Li Y, Zhang LJ, Wei Y, et al Genetic diversity of a Chinese native chicken breed, Bian chicken, based on twenty-nine microsatellite markers. Asian-Australasian J Anim Sci. 2010;23: 154–161. 10.5713/ajas.2010.90367

[20] Ashley M V, Dow BD. The use of microsatellite analysis in population biology: background, methods and potential applications. EXS. 1994 pp. 185–201. 10.1007/978-3-0348-7527-1_10 7994106

[21] Pandey AK, Tantia MS, Kumar D, Mishra B, Chaudhary P, Vijh RK. Microsatellite Analysis of Three Poultry Breeds of India. Asian-Australasian J Anim Sci. 2002;15: 1536–1542. 10.5713/ajas.2002.1536

[22] Bruford MW, Wayne RK. Microsatellites and their application to population genetic studies. Curr Opin Genet Dev. Elsevier Current Trends; 1993;3: 939–943. 10.1016/0959-437x(93)90017-j 8118220

[23] Li YC, Korol AB, Fahima T, Beiles A, Nevo E. Microsatellites: Genomic distribution, putative functions and mutational mechanisms: A review. Molecular Ecology. 2002 pp. 2453–2465. 10.1046/j.1365-294x.2002.01643.x 12453231

[24] Putman AI, Carbone I. Challenges in analysis and interpretation of microsatellite data for population genetic studies. Ecol Evol. 2014;4: 4399–4428. 10.1002/ece3.1305 25540699 PMC4267876

[25] Gärke C, Ytournel F, Bed’Hom B, Gut I, Lathrop M, Weigend S, et al Comparison of SNPs and microsatellites for assessing the genetic structure of chicken populations. Anim Genet. 2012;43: 419–428. 10.1111/j.1365-2052.2011.02284.x 22497629

[26] DA D, AD B, LG S, D M-G, IR S, GJ H, et al High-utility conserved avian microsatellite markers enable parentage and population studies across a wide range of species. BMC Genomics. 2013;14: 176 Available: http://ovidsp.ovid.com/ovidweb.cgi?T=JS&CSC=Y&NEWS=N&PAGE=fulltext&D=medl&AN=23497230 10.1186/1471-2164-14-176 23497230 PMC3738869

[27] Claire DH, Donald RK, Aline K, Tiba M, Fabrice S, Gaspard U, et al Phenotypes, production systems and reproductive performance of indigenous chickens in contemporary Rwanda. Int J Livest Prod. 2019;10: 213–231. 10.5897/IJLP2019.0618

[28] Clay DC, Dejaegher YMJ. Agro-ecological Zones: The Development of a Regional Classification Scheme for Rwanda [Internet]. Tropicultura. 1987 pp. 153–159. Available: http://www.tropicultura.org/text/v5n4/153.pdf

[29] Mwambene PL, Kyallo M, Machuka E, Githae D, Pelle R. Genetic diversity of 10 indigenous chicken ecotypes from Southern Highlands of Tanzania based on Major Histocompatibility Complex-linked microsatellite LEI0258 marker typing. Poult Sci. 2019;98: 2734–2746. 10.3382/ps/pez076 30877744 PMC6591683

[30] Smith L, Burgoyne L. Collecting, archiving and processing DNA from wildlife samples using FTA ® databasing paper. BMC Ecol. BioMed Central; 2004;4: 4 10.1186/1472-6785-4-4 15072582 PMC406513

[31] ISAG/FAO Standing Committee. Secondary Guidelines for Development of National Farm Animal Genetic Resources Management Plans. Measurement of Domestic Animal Diversity (MoDAD): Recommended Microsatellite Markers [Internet]. Rome; 2004 Available: http://www.fao.org/3/a-aq569e.pdf

[32] Fao. Molecular genetic characterization of animal genetic resources. FAO Animal Production and Health Guidlines 2011.

[33] Vanhala T, Tuiskula-Haavisto M, Elo K, Vilkki J, Maki-Tanila A. Evaluation of genetic variability and genetic distances between eight chicken lines using microsatellite markers. Poult Sci. Oxford University Press; 1998;77: 783–790. 10.1093/ps/77.6.783 9628523

[34] Zhu WQ, Li HF, Wang JY, Shu JT, Zhu CH, Song WT, et al Molecular genetic diversity and maternal origin of Chinese black-bone chicken breeds. Genet Mol Res. 2014;13: 3275–3282. 10.4238/2014.April.29.5 24841659

[35] Berthouly C, Leroy G, Van TN, Thanh HH, Bed’Hom B, Nguyen BT, et al Genetic analysis of local Vietnamese chickens provides evidence of gene flow from wild to domestic populations. BMC Genet. BioMed Central; 2009;10: 1 10.1186/1471-2156-10-1 19133138 PMC2628941

[36] Mohammadabadi MR, Nikbakhti M, Mirzaee HR, Shandi a., Saghi D a., Romanov MN, et al Genetic variability in three native Iranian chicken populations of the Khorasan province based on microsatellite markers. Russ J Genet. 2010;46: 505–509. 10.1134/S1022795410040198 20536031

[37] Chen G, Bao W, Shu J, Ji C, Wang M, Eding H, et al Assessment of population structure and genetic diversity of 15 Chinese indigenous chicken breeds using microsatellite markers. Asian-Australasian J Anim Sci. 2008;21: 331–339.

[38] Eltanany M, Philipp U, Weigend S, Distl O. Genetic diversity of ten Egyptian chicken strains using 29 microsatellite markers. Anim Genet. 2011;42: 666–669. 10.1111/j.1365-2052.2011.02185.x 22035011

[39] Nadeem A, Sciences A, Hussain T, Wajid A, Shah S, Sciences A. Microsatellite Marker Based Genetic Diversity among Four Varieties of Pakistani Aseel Chicken. Pak Vet J. 2011; 2074–7764.

[40] Cuc NTK, Muchadeyi FC, Baulain U, Eding H, Weigend S, Wollny CBA. An assessment of genetic diversity of Vietnamese H’mong chickens. Int J Poult Sci. 2006;5: 905–913. 10.3923/ijps.2006.905.911

[41] Clementino CS, Barbosa FJ V, Carvalho AMF, Costa-Filho RAR, Silva GR, Campelo EG, et al Microsatellite DNA Loci for poulation studies in Brazilian chicken ecotypes. Int J Poult Sci. 2010;9: 1100–1106.

[42] Dana N. Breeding programs for indigenous chicken in Ethiopia: analysis of diversity in production systems and chicken populations [Internet]. 2011 Available: http://library.wur.nl/WebQuery/wda/lang/1955246

[43] Cheng H-W. Breeding of tomorrow’s chickens to improve well-being1. Poult Sci. 2010;89: 805–813. 10.3382/ps.2009-00361 20308415

[44] Kaya M, Yildiz MA. Genetic diversity among Turkish native chickens, Denizli and Gerze, estimated by microsatellite markers. Biochem Genet. Springer; 2008;46: 480–91. 10.1007/s10528-008-9164-8 18500550 PMC2480610

[45] Getu A, Tadesse A. A Phenotypic and Genetic Characterized Indigenous Chicken Ecotypes in Ethiopia. Int J Genet. 2014;2014: 4–10. 10.5829/idosi.ijg.2014.4.1.8523

[46] Dana N, van der Waaij LH, Dessie T, van Arendonk JAM. Production objectives and trait preferences of village poultry producers of Ethiopia: Implications for designing breeding schemes utilizing indigenous chicken genetic resources. Trop Anim Health Prod. 2010;42: 1519–1529. 10.1007/s11250-010-9602-6 20512411 PMC2929342

[47] Mwacharo JM, Nomura K, Hanada H, Jianlin H, Hanotte O, Amano T. Genetic relationships among Kenyan and other East African indigenous chickens. Anim Genet. 2007;38: 485–490. 10.1111/j.1365-2052.2007.01641.x 17894562

[48] Lyimo, C.M, Weigend, A., Janßen-Tapken, U., Msoffe, P.L., Simianer, H. and Weigend S. A Global Assessment of Population Structure and Genetic Diversity in Chicken Populations from Africa, Asia, Europe, Red Jungle Fowls and Commercial Breeds. Tropentag 2013, Stuttgart, Germany September 17–19, 2013. 2013. pp. 1–4. Available: http://www.tropentag.de/2013/abstracts/full/551.pdf

[49] Boettcher PJ, Tixier-Boichard M, Toro MA, Simianer H, Eding H, Gandini G, et al Objectives, criteria and methods for using molecular genetic data in priority setting for conservation of animal genetic resources. Anim Genet. 2010;41: 64–77. 10.1111/j.1365-2052.2010.02050.x 20500756

[50] Kuan-Wei C, Ya-Bo Y, Jin-Yu W, Mekki DM, Qing-Ping T, Hui-Fang L, et al Evaluation of Genetic Diversity and Genetic Distance Between Twelve Chinese Indigenous Chicken Breeds Based on Microsatellite Markers. Int J Poult Sci. 2006;5: 550–556. 10.3923/ijps.2006.550.556

[51] Laval G, SanCristobal M, Chevalet C. Measuring genetic distances between breeds: use of some distances in various short term evolution models. Genet Sel Evol. 2002;34: 481–507. 10.1186/1297-9686-34-4-481 12270106 PMC2705457

[52] Rischkowsky B, Pilling D. The State of the World’s Animal Genetic Resources for Food and Agriculture [Internet]. 2007 http://www.fao.org/3/a-i4787e/index.html

[53] Wilkinson S, Wiener P, Teverson D, Haley CS, Hocking PM. Characterization of the genetic diversity, structure and admixture of British chicken breeds. Anim Genet. 2012;43: 552–563. 10.1111/j.1365-2052.2011.02296.x 22497565

[54] Peripolli E, Munari DP, Silva MVGB, Lima ALF, Irgang R, Baldi F. Runs of homozygosity: current knowledge and applications in livestock. Animal Genetics. 2017 pp. 255–271. 10.1111/age.12526 27910110

[55] Hassen H, Neser FWC, Kock A De. Study on the genetic diversity of native chickens in northwest Ethiopia using microsatellite markers. J Biotechnol. 2009;8: 1347–1353.

[56] Nigussie H, Kebede K, Ameha N. Phenotypic and Morphological Characterization of Indigenous Chicken Populations in Southern Zone of Tigray, Ethiopia. J Biol. 2015;5: 132–141. Available: www.iiste.org

[57] Ngeno K, van der Waaij EH, Megens HJ, Kahi AK, van Arendonk JAM, Crooijmans RPMA. Genetic diversity of different indigenous chicken ecotypes using highly polymorphic MHC-linked and non-MHC microsatellite markers. Anim Genet Resour génétiques Anim genéticos Anim. 2015;56: 1–7. 10.1017/S2078633614000484

[58] MINAGRI. Strategy and Investment Plan to strengthen the animal genetic improvemen in Rwanda. 2012.

