# Correction: Genetic diversity and population structure of indigenous chicken in Rwanda using microsatellite markers

**DOI:** 10.1371/journal.pone.0238966

**Published:** 2020-09-03

**Authors:** Richard Habimana, Tobias Otieno Okeno, Kiplangat Ngeno, Sylvere Mboumba, Pauline Assami, Anique Ahou Gbotto, Christian Tiambo Keambou, Kizito Nishimwe, Janvier Mahoro, Nasser Yao

There is an error in affiliation 6 for Christian Tiambo Keambou. The correct affiliation is: Centre for Tropical Livestock Genetics and Health–International Livestock Research Institute.
