# Supplementary material for: Genetic diversity and population structure of indigenous chicken in Rwanda using microsatellite markers
Source: PLoS One. 2020 Apr 2;15(4):e0225084. doi: 10.1371/journal.pone.0225084 (PMC7117670; doi:10.1371/journal.pone.0225084)
Supplement: S1 Table — (DOCX) [file pone.0225084.s001.docx]

| **Characteristics** | **Indigenous chicken(IC)^1^** | **Kuroiler^2^** | **Cobb^3^** | **ISA Brown^4^** |
| --- | --- | --- | --- | --- |
| Origin | not yet determined | India | USA | France |
| Development year | N/A | 1990 | 1916 | 1978 |
| Comb | single | single | single | single |
| Main color | multi-coloured | several color varieties | white | Brown |
| Purpose | dual purpose( meat and egg) | meat and egg) | Meat purpose | Egg purpose |
| Size | light | large | large | Light |
| Cock weight | 1.8 kg in 300 days | 4-5 kg in 150-180 days | 1.9 kg in 35 days | 2.72 kg |
| Cockrel weight | 1.3 kg in 200-250 days | 2.5-3.5 kg |  | 1.80 kg |
| Hen weight | 1.5 kg in 300 days | 2.5 kg | 2.4 kg in 42 days | 2 kg |
| Pullet weight | 1 kg in 200-250 days | 1.8 kg |  | 1.5 kg |
| Egg capacity per year | 60-100 | 150-200 | N/A | 300 |
| Age at first Egg (days) | 210 | 150 | N/A | 120-130 |
| Egg weight ( g) | 45-50 | medium | N/A | 60 |
| Egg size | small | medium | N/A | Extra Large |
| Egg color | white | light brown | N/A | Brown |
| Skin color | white | white | yellow | Yellow |
| Shank color | mainly yellow | yellow to blackish | yellow | Yellow |
| Survivability %  upto 6 weeks | 100% | 99% | 92-93% | 98 |

**S1 Table. Characteristics of indigenous and exotic chickens used in the study**

J. Mahoro, T. K. Muasya, F. Mbuza, R. Habimana, and A. K. Kahi, “Characterization of indigenous chicken production systems in Rwanda,” *Poult. Sci.*, vol. 96, no. 12, pp. 4245–4252, Dec. 2017 and D. H. Claire *et al.*, “Phenotypes, production systems and reproductive performance of indigenous chickens in contemporary Rwanda,” *Int. J. Livest. Prod.*, vol. 10, no. 10, pp. 213–231, Dec. 2019

2 <https://value.co.ke/article/kuroiler-chicken-farming-breed-information-and-management>

3 <https://www.cobb-vantress.com/en_US/products/>

4 <https://domesticanimalbreeds.com/isa-brown-chicken-breed-everything-you-need-to-know/>
