# Supplementary figures and images for: Genetic diversity and population structure of indigenous chicken in Rwanda using microsatellite markers
Source: PLoS One. 2020 Apr 2;15(4):e0225084. doi: 10.1371/journal.pone.0225084 (PMC7117670; doi:10.1371/journal.pone.0225084)

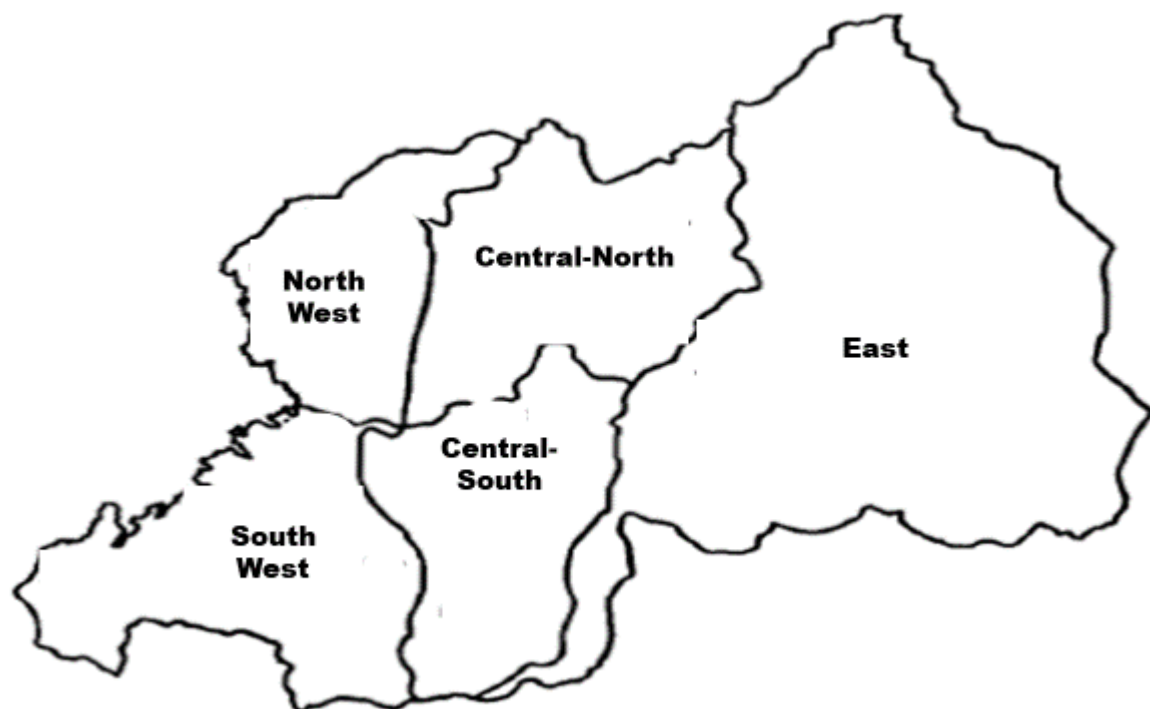

**S2\_Fig. Agro ecological zones in Rwanda**

Source: Clay and Dejaegher, 1987

Supplement: S1 Fig — (PDF) [file pone.0225084.s002.pdf]
