# Supplementary material for: Genetic diversity and population structure of indigenous chicken in Rwanda using microsatellite markers
Source: PLoS One. 2020 Apr 2;15(4):e0225084. doi: 10.1371/journal.pone.0225084 (PMC7117670; doi:10.1371/journal.pone.0225084)
Supplement: Attachment — Submitted filename: Response to Reviewers.docx [file pone.0225084.s003.docx]

| **Editor & Reviewers** | **Points raised**  **Matrix of the responses to the academic editor and reviewers** | **Response** |
| --- | --- | --- |
| Academic editor | A rebuttal letter that responds to each point raised by the academic editor and reviewer(s) | Done |
|  | A marked-up copy of your manuscript that highlights changes made to the original version | Done |
|  | An unmarked version of your revised paper without tracked changes | Done |
|  | Please amend your current ethics statement to confirm that your named ethics committee specifically approved this study | Ethic statement amended |
|  | Once you have amended this/these statement(s) in the Methods section of the manuscript, please add the same text to the “Ethics Statement” field of the submission form (via “Edit Submission”). | Amended ethical clearance has been put in the methods section and also added to the Ethics Statement field of the submission form |
|  | In your Methods section, please provide additional details regarding the chicken used in your study and ensure you have described the source. | Additional details regarding the chicken used in this study have been added in the supporting information (**S1 Table**). |
|  | In your Methods section, please provide additional location information of the sampling locations, including geographic coordinates for the data set if available | Additional information of the sampling locations, including geographic coordinates for the data set were provided in **S2 Fig and Fig 1.** |
|  | In your Methods section, please provide additional information regarding the permits you obtained for the work. Please ensure you have included the full name of the authority that approved the sampling locations access and, if no permits were required, a brief statement explaining why | Additional information regarding the permits I obtained for the work has been provided and the full name of the authority that approved the sampling locations access was given |
| **Reviewer # 1** | The writing of the introduction is poor. The introduction section only introduces the importance of indigenous chicken, and I suggest adding some information about the recent research of microsatellite markers in chickens | Introduction has been improved and some information on microsatellite markers has been included in the introduction section (line 61-72) |
|  | In the materials and methods, the twelve exotic chickens (layers and broilers) as a reference data should have a detailed information. | Detailed information on exotic chicken has been discussed and more details were put in supporting information (**S1 Table**). |
|  | Statistical analysis methods are not clearly presented, for example, p-value is mentioned without description of what kind of test. In addition, the p-value should be written consistently in the all manuscript. | Statistical analysis has been improved.  Concerning tests for the Hardy-Weinberg Equilibrium (HWE), P-value was obtained based on chi square (χ2) test and the nonconformity with HWE based on Chi square test at p<0.05.  In addition, p-value has been written consistently throughout the manuscript. |
|  | Line 31: “chicken” should be “chickens” | Corrected🡪 line 26 |
|  | Line 243: “3,31” should be “3.13”. | Corrected🡪 line 307 |
|  | Line 243: “drift-” should be “drift”. | Corrected 🡪 line 350 |
|  | Please carefully check the format of the manuscript, and make sure it fits the journal style | The manuscript now fits the journal format |
| **Reviewer #2** | **Introduction:** The authors should reformulate the introduction in order to improve the understanding. For example, I could not understand the sentence: “More than 40% of households keep poultry out of which approximately 80% consists of indigenous chicken (IC).” (lines 50-51). | The introduction has been reformulated as per your request and it is now understandable. That confusing sentence has been paraphrased and it is now “ **More than 40% of households keep poultry with indigenous chickens being the most preferred, accounting for approximately 80% of the reared chicken species”** |
|  | **M&M:** Please, specify with more details sample the collection method and the ethical permission for collection. The sentences describing the genetic groups should contain only the breed´s name, number of samples used and also, the number of regions/flocks that samples were collected. I suggest that description of phenotypic traits in a separate table, for better understanding | The collection method has been detailed and ethical statement included.  The genetic groups were named based on the agro-ecological zone of origin. In total, 313 IC were sampled from five agro-ecological zones (**S2 Fig**) and in each region, a specific number of IC was collected according to the size of the region.  Phenotypic data was provided in supporting information (**S1 Table**) |
|  | **Discussion:** In general, the discussion is basically descriptive, improving the information of the results previously showed and comparing the results of this and other papers already published. There is no discussion in terms of phylogenetic relationships or evolution, neither about the genetic distance within the breeds studied, which weakens the impact of the paper. The manuscript subject is interesting, but needs to be improved  The English written should be improved | You are right. The discussion on phylogenetic relationship or evolution was under the subtitle called **population structure**; that is why it did not come out clearly. So as to make it clear, apart from its improvement, the subtitle has also been changed into “**Phylogenetic relationship and Population structure**”.  The discussion on genetic distance is under “genetic relationship subtitle” and it has been improved as per your request.  The English written has been improved as you can see that thru the track changes. |
